# High Performance Mixed Potential Type NO_2_ Gas Sensor Based on Porous YSZ Layer Formed with Graphite Doping

**DOI:** 10.3390/s19153337

**Published:** 2019-07-30

**Authors:** Hao Hong, Jianwen Sun, Cinan Wu, Zewen Liu

**Affiliations:** 1 College of Big Data and Information Engineering, Guizhou University, Guiyang 550025, China; 2 Department of Microelectronics, Delft University of Technology, 2628 CD Delft, The Netherlands; 3 China Research Institute, Delft University of Technology, Beijing 100083, China; 4 Institute of Microelectronics, Tsinghua University, Beijing 100084, China

**Keywords:** mixed potential, NO_2_, porous YSZ layers, graphite

## Abstract

High performance mixed potential type NO_2_ sensors using porous yttria-stabilized zirconia (YSZ) layers doped with different concentration graphite as solid electrolyte and LaFeO_3_ as sensing electrode were fabricated and characterized. LaFeO_3_ was prepared by a typical citrate sol–gel method and characterized using XRD. The surface morphology and porosity of porous YSZ layers were characterized by field emission scanning electron microscope (FESEM). The sensor doped with 3 wt% graphite shows the highest response (−76.4 mV to 80 ppm NO_2_) and the response is linearly dependent on the logarithm of NO_2_ concentration in the range of 10–200 ppm. The sensor measurement results also present good repeatability and cross-sensitivity.

## 1. Introduction

With the accelerated development of automobiles and industry, gas emission has caused serious environmental disasters and human diseases. Among the emitted gas, nitrogen dioxide (NO_2_) is the most dangerous [1,2,3]. In order to detect and monitor the NO_2_ precisely, high-performance NO_2_ sensors are in high demand [4,5,6].

Since Fleming first observed non-Nernst behavior in 1977, the mixed potential theory has been proposed consequently [7]. Mixed potential type gas sensors consist of a high-temperature stable electrolyte, a porous sensitive electrode (SE), and a reference electrode (RE). It could be used to detect various target gases if the sensitive material used properly [8]. Currently, the most suited electrolyte for those sensors is yttria-stabilized zirconia (YSZ) due to its excellent chemical and thermal stability at high temperatures. Comparing to the metal oxide semiconductor, catalytic combustion and surface acoustic wave (SAW) gas sensors, the mixed type potential sensor has many advantages including better mechanical properties, chemical stability and thermal stability at high temperatures [9], which make mixed potential sensor work well in the harsh environment such as the automobile exhaust gas after-treatment system [8,10,11,12,13]. Recently, to further improve the performance of the mixed potential sensor effectively, many groups have turned their attention to finding more sensitive materials including single metal oxides, composite perovskite and spinel-type oxides, and also made some good results [14,15]. Researchers used In_2_O_3_ as SE had reached a NO_2_ response of 126 mV to 100 ppm at 700 °C [16]. Liu et al. reported a mixed potential sensor based on Pt/YSZ/CoTa_2_O_6_ displays segmentally linear relationship to the logarithm of NO_2_ concentration in the ranges of 0.5–5 ppm and 5–500 ppm, with sensitivities of 12 and 80 mV/decade at 650 °C, respectively [17]. These sensors exhibit high response and good sensitivity to NO_2_. It demonstrates the used materials’ good catalytic properties to NO_2_. However, the operating temperature of the proposed sensor is above 600 °C, which may not match the low-temperature region of an exhaust pipe. However, according to the mixed potential type sensing mechanism, the performance of the mixed potential sensor is not only related to the catalytic ability of the sensitive material, but also the structure of the triple-phase boundary (TPB) and a larger TPB means more active sites for the electrochemical reaction. In recent years, research works are focused on increasing the TPB by constructing YSZ substrates such as hydrofluoric acid corrosion, sand lasting, laser fabrication and ion beam etching, and mixing YSZ with sensitive material to obtain the larger TPB [18,19,20,21,22]. Due to these proposed technologies, the TBP increases effectively. However, some of those methods like mixing YSZ with sensitive material, which causes the covering of the electrochemical reaction sites and resulting in a low response. Other methods such as using high-cost ion beam etching need complex processes and make the experiment difficult to be repeated. Moreover, most of the mixed potential sensors usually work at 300–800 °C, while it is also desirable for a high-performance sensor with low operating temperatures such as the vehicle exhaust pipe (200–300 °C). Those sensors may not work properly or result in low performance at this temperature. A NO_2_ sensor located downstream of the exhaust pipe should operate at a similar temperature as the exhaust gases (200–300 °C). Otherwise, an additional heating unit may be necessary and it would take more power assumption and cost.

In this work, we report a high performance mixed potential type NO_2_ sensor using the porous YSZ layer. The porous YSZ layer was prepared by adding graphite to YSZ paste, and screen-printing to the initial YSZ substrate. To the best of our knowledge, there are no reports about this low-cost and facile method. Due to the larger TPB and uniform porous layer, the fabricated sensor response to NO_2_ improves. The presented mixed potential NO_2_ sensor doped with 3 wt% graphite exhibits the highest performance and response to 80 ppm NO_2_ is −76.4 mV. Moreover, the sensor also exhibits excellent repeatability during continuous five cycles’ measurement and good cross-sensitivity compared with acetone, CO and CH_4_. Additionally, the fabricated sensor optimum operating temperature is 250 °C. Comparing to existing publications [16,17,18], the working temperature of the presented sensor is lower than most of the publications. This high performance mixed potential type NO_2_ sensor could be installed on an exhaust pipe without extra heating units and could monitor the NO_2_ emission in the low-temperature region. In the following session, the experiment on sensor material preparation, device fabrication and the detailed investigation of the properties of the fabricated sensor are presented.

## 2. Experiment

### 2.1. Material

The LaFeO_3_ powder was prepared by a typical citrate sol–gel method, and all the raw materials were of analytic grade. At first, appropriate La(NO_3_)_3_·6H_2_O, Fe(NO_3_)_3_·9H_2_O, citric acid and ethylene glycol were dissolved in deionized water with molar ratios of 1:1:2:6. Then the solution was stirred for 4 h at 80 °C to obtain the sol precursor. The sol precursor was dried for 24 h at 120 °C and then we got a gel precursor, after that the gel precursor was pre-calcined at 400 °C to gain LaFeO_3_ precursor powder. Finally, the precursor was ground with an agate mortar and then calcined for 2 h at 700 °C to acquire the LaFeO_3_ powder. 

Porous YSZ paste was fabricated by a simple stirring method. At first, different concentration graphite (0 wt%, 3 wt%, 10 wt%, 15 wt%) was added to the YSZ powder (8 mol Y_2_O_3_-doped). Then the mixture was dissolved in the colloid adhesive, which was prepared by mixing α-terpineol, cellulose dispersant, leveling agent and defoamer. In the next step the vibration tumbling machine was used to stir the obtained precursor paste to acquire the porous YSZ paste. Finally, porous YSZ paste was deposited on the initial YSZ substrate by the screen-printing method, after calcination the porous YSZ layer was formed. 

### 2.2. Fabrication of the Sensor

**Figure 1:**
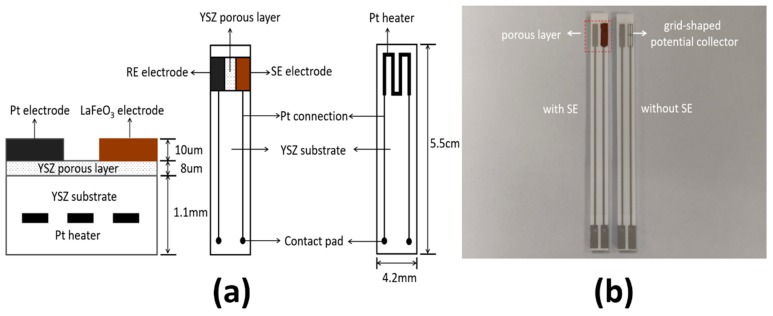
(**a**) Schematic structure and (**b**) the top view of the sensor.

As shown in Figure 1, the mixed potential planar sensor with the configuration of LaFeO_3_/YSZ/Pt was fabricated by the screen-printing method. Firstly, the Pt heater was printed in the initial YSZ substrate (5.5 cm × 4.2 mm × 1.1 mm), after calcination, the porous YSZ paste was printed on the initial YSZ substrate mentioned before and then calcination. Graphite was oxidized to CO_2_ at 750 °C in this process. After that a stripe-shaped and a grid-shaped Pt electrode (5 mm × 1 mm) were performed on the two sides of the porous YSZ layer using commercial Pt paste (Pt-JC, electroplating engineers of Japan, Ltd., Hiratsuka, Japan) as the RE and potential collector. At the same time the Pt connection was also printed on the substrate. After calcination, the SE paste was obtained by mixing LaFeO_3_ powder with the appropriate colloid adhesive. Then the obtained paste was deposited on the grid-shaped Pt electrode. The distance between the two electrodes was 1 mm. Finally, the sensor was sintered again at the temperature of 1200 °C to get a dense structure. The fabricated sensors using the porous YSZ layers with different concentration graphite (0 wt%, 3 wt%, 10 wt%, 15 wt%) were labeled as Sensor A, Sensor B, Sensor C and Sensor D.

### 2.3. Measurement of the Sensor

X-ray (Rigaku SmartLab., Tokyo, Japan) diffraction with Cu-Kα radiation was used for analyzing the crystalline structure of the LaFeO_3_ powder. The surface morphology and porosity of the porous YSZ layers were characterized by field emission scanning electron microscope (FESEM; FEI Company, Brno, Czech Republic). The gas sensing performance of the sensor was measured by a conventional static method. The gas sensing experiment was carried out in a clean room with humidity of 56% and environment temperature of 26 °C, with an oxygen concentration of 20.8% in a standard atmosphere. The heating voltage was provided by a DC power supply (6 V-250 °C, 7 V-300 °C, 8 V-350 °C, 9 V-400 °C and 10 V-450 °C) and an infrared thermometer was used to monitor the surface of the device in real time. The potential response was measured by a digital multimeter (Keithley 2400). The sample gas with 10–200 ppm NO_2_ was prepared by diluting 2000 ppm NO_2_ standard gas by air. The gases for cross-sensitivity measurement including CO, CH_4_ were prepared by diluting 4000 ppm CO, CH_4_ standard gas by air and acetone was diluting with volume ratio. According to the mixed potential theory, other carrier gases were unnecessary. The detailed gas sensing measurement was as follows. At first, the sensor was placed in an airtight chamber with a volume of 500 mL. Next the gas in the chamber was pumped out and then fresh air was pumped in. Before injecting the target gas into the chamber, using DC power to supply voltage when the sensor’s temperature was stable. Then turning off the pump and a certain amount of gas was injected into the chamber via the different sizes of needles (5 mL, 10 mL and 50 mL) in a few seconds. After 400 s, when the response magnitude was almost unchanged, turning on the pump for another 400 s, the rest of the gas was pumped out and the pure air filled the chamber. That was one cycle measurement.

## 3. Result and Discussion

**Figure 2:**
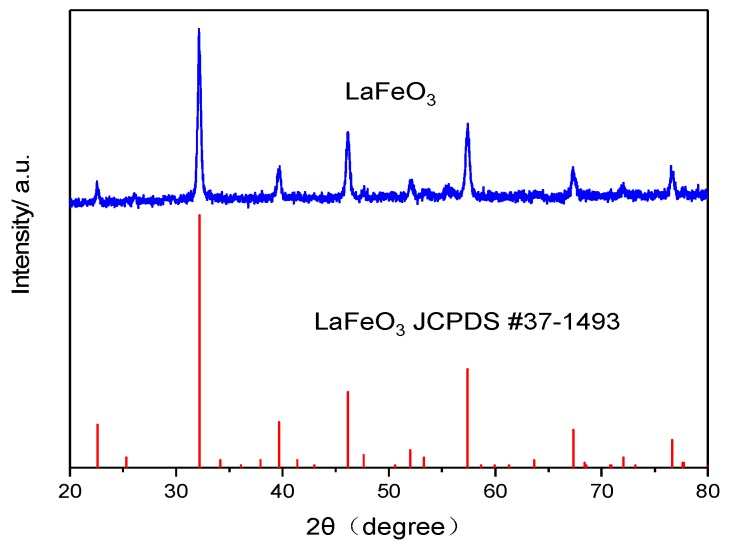
XRD patterns of LaFeO_3_ sensing electrode material.

**Figure 3:**
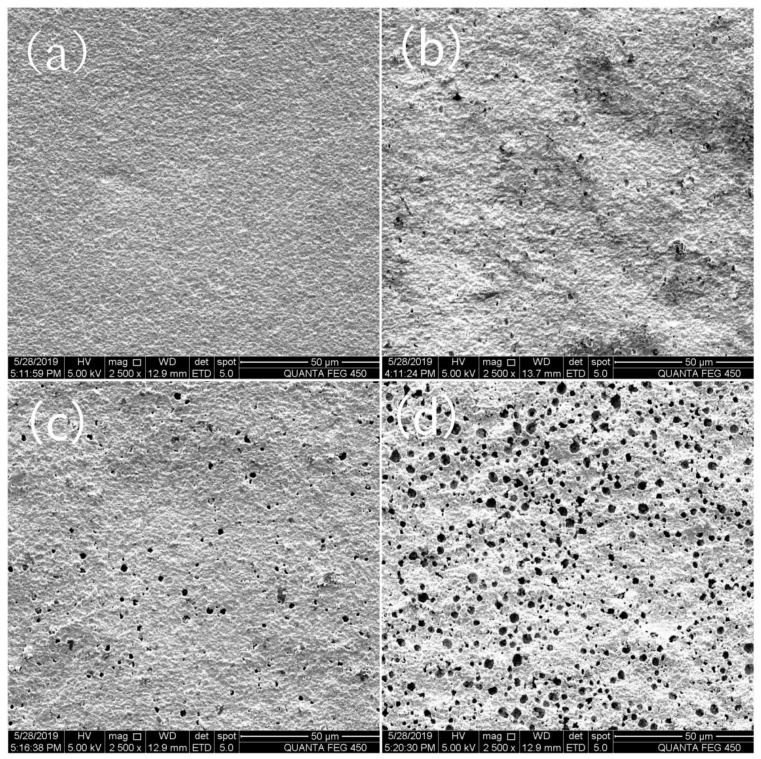
Field emission scanning electron microscope (FESEM) graph of the fabricated yttria-stabilized zirconia (YSZ) porous layer with different doping (**a**) 0 wt%, (**b**) 3 wt%, (**c**) 10 wt% and (**d**) 15 wt%.

Figure 2 shows the X-ray diffraction (XRD) pattern of the LaFeO_3_ powder prepared by a typical citrate sol–gel method, the result demonstrated that all diffraction peaks of the LaFeO_3_ powder were in correspondence with the standard LaFeO_3_ patterns of JCPDS card No.37-1493, No other diffraction peaks of the sensitive material were observed, which indicates the high purity of the LaFeO_3_. The surface morphology and porosity of sensors were characterized by FESEM. Figure 3 are the SEM images of porous YSZ layer fabricated with 0 wt%, 3 wt%, 10 wt% and 15 wt% graphite. It could be clearly seen that few cavities were found on the YSZ layer without any graphite (Figure 3a). However, there was a large number of holes on the surface when the graphite doping in. With the increase of the graphite concentration, more holes could be found on the porous layer and the size of holes was becoming larger in Figure 3b–d, averagely 2.2 μm, as shown in Figure 3d.

**Figure 4:**
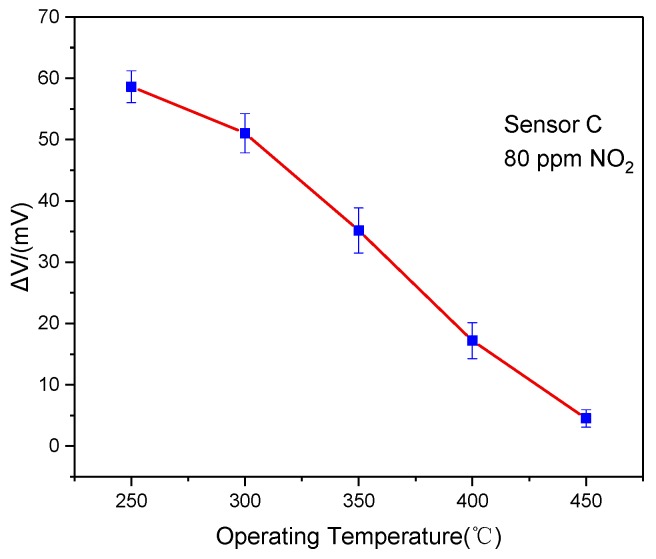
Response changes of the sensor to 80 ppm NO_2_ at a different operating temperature.

It is known that the performance of the mixed potential sensor relies on the operating temperature. In order to investigate the optimum operating temperature, the response of Sensor C was measured in the temperature at the range of 250–450 °C. As demonstrated in Figure 4, it was apparent that the response decreased with the increasing operating temperature. It was found the maximum response to 80 ppm NO_2_ was −58.6 mV at 250 °C. The reason for this phenomenon might be explained as follows. The definite activation energy was a necessary condition for the electrochemical reaction in TPB. However, the desorption process of NO_2_ became dominant at above 250 °C, and the amount of NO_2_ adsorbed on LaFeO_3_ was less along with the increasing temperature. Hence, the response of the sensor to NO_2_ decreased with further increasing temperature [19,23,24,25].

Table 1 shows a comparison of the mixed potential type NO_2_ sensor’s operating temperature with different materials and configurations. The operating temperature of the presented sensor was the lowest. Due to catalytic properties of the LaFeO_3_, comparing with other sensitive materials including single and spinel oxides, the sensor with LaFeO_3_/YSZ/Pt exhibited a lower operating temperature. On account of the uniform porous YSZ layer, the fabricated sensor with LaFeO_3_/YSZ/Pt worked at a lower temperature than the sensors with the same sensitive material and configuration. It indicated a low power assumption of the presented sensor.

**Figure 5:**
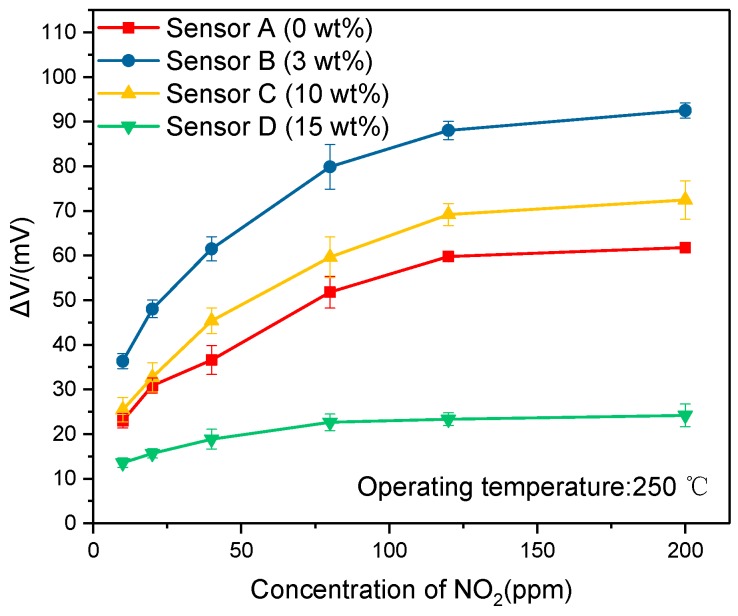
Response of the sensors doped with 0 wt%, 3 wt%, 10 wt% and 15 wt% to 10–200 ppm NO_2_ at 250 °C.

Figure 5 shows the response of the sensors doped with different concentration graphite, which means the different sizes and density of holes on the porous layer exposure to 10–200 ppm of the test gas. It was found that the Sensor B doped with 3% graphite exhibited the highest value and the response to 80 ppm NO_2_ was −76.4 mV. It proved the function of the YSZ porous layer, which increased the contact area between SE and YSZ substrate effectively. That could be understood there were more active sites for the electrochemical reaction and then the output potential of sensor increased. However, overfull graphite made the YSZ porous layer lower adhesion. Hence the YSZ porous layer could not combine both with SE and RE well, which caused an increase in interface resistance, and resulted in the lower response. The result also means the existing of an optimizing doping concentration. In order to investigate the detailed relationship between NO_2_ concentration and the response, transient response and recovery characteristics of Sensor C to different concentration of NO_2_ were researched.

**Figure 6:**
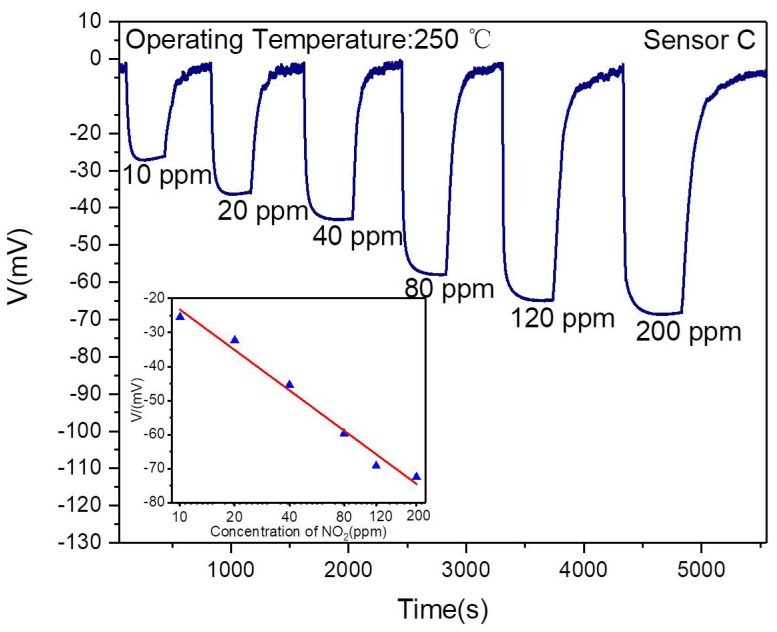
Transient response and recovery characteristics of Sensor C to different concentrations of NO_2_ at 250 °C.

As illustrated in Figure 6. It was found that the response increased with the NO_2_ concentration increasing. It could be understood that the response of the fabricated sensor was determined by the number of NO_2_ molecules in TPB. When the NO_2_ concentration increases, there would be more NO_2_ arriving at TPB to participate in the electrochemical catalytic reaction and resulting in a higher response. From this figure, the response was linearly dependent on the logarithm of NO_2_ concentration in the range of 10–200 ppm, which was following the sensing mechanism according to the mixed potential theory. It could also be explained by Equation (1), which clearly shows the response of the sensor linearly varies with the NO_2_ concentration [34].

V = A − BlnC_NO2_, (1)

where V is the response, A and B are constants and C_NO2_ is the concentration of NO_2_. The increase of the sensor response to 80–200 ppm NO_2_ is lower than that of 10–80 ppm, which could also be demonstrated in Figure 5. It can be speculated that when the number of adsorbed molecules increases, desorption of the molecules also enhances. Finally, the rates of the two processes were equal and the electrochemical reactions of the fabricated sensors reached saturation slowly, which is similar to the phenomena described in Langmuir’s theory [35]. The response and recovery time were studied in the next part.

**Figure 7:**
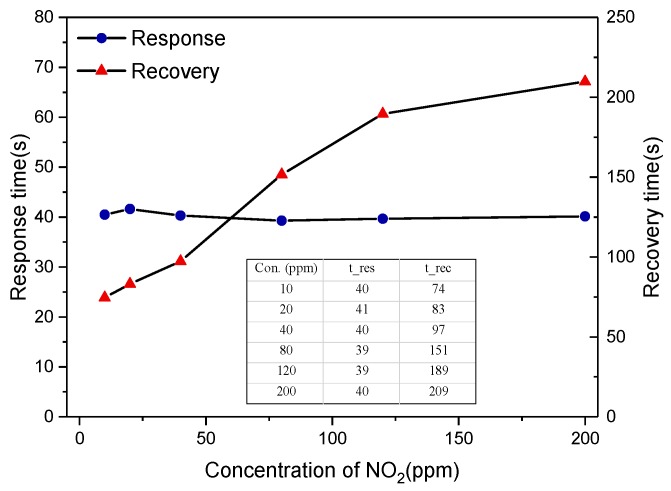
Response and recovery time of Sensor C to different concentrations of NO_2_.

Deriving from the experiment evidence based on Figure 6, Figure 7 gives an insight into the response and recovery time. It exhibits that the response time was basically stable at 40 s and recovery time increased with the increasing of NO_2_ concentration, and the recovery time to 200 ppm NO_2_ was 209 s. It could be explained that it would take more time for the process of desorption because of the increasing NO_2_ concentration, lower temperature. Another reason was the slow pumping speed in the experiment, while that in the automobile exhaust pipe was very fast. The presented sensor could be a potential candidate for practical automobile exhaust application.

**Figure 8:**
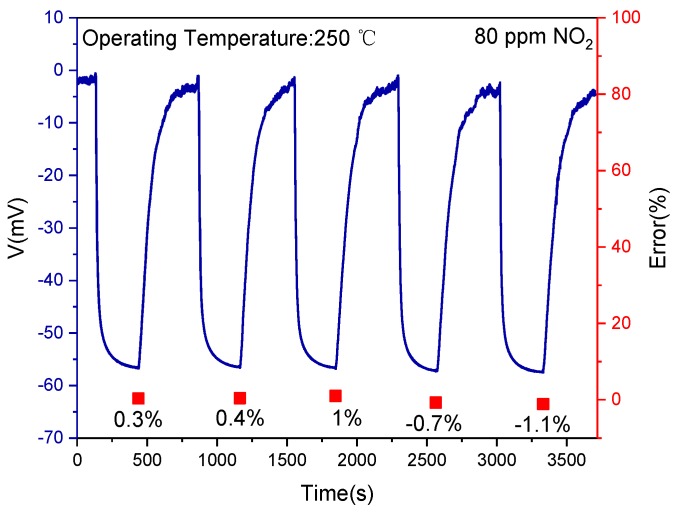
Continuous five times response–recovery transient characteristics of Sensor C to 80 ppm NO_2_ at 250 °C.

**Figure 9:**
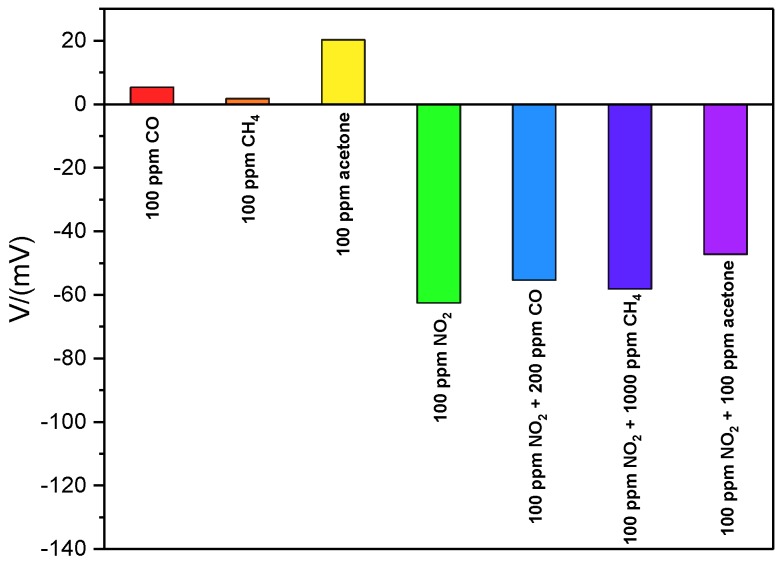
Cross-sensitivity measurement for Sensor C to 100 ppm various gases at 250 °C.

To study the error in the response of the fabricated sensor, Figure 8 shows the continuous five cycles’ measurement of Sensor C to 80 ppm NO_2_ gas at 250 °C. The error of response is denoted by (V − V_0_)/V_0_ × 100%, where V and V_0_ represent the sensor response and the average of the five responses, respectively. It is seen that the response and recovery characteristics of the presented sensor held good consistency during the continuous five cycles’ measurement and the maximum error of the response to 80 ppm NO_2_ was −1.1%, which demonstrated good repeatability of the sensor. Cross-sensitivity for Sensor C to 100 ppm of various gases including CH_4_, CO, and acetone was tested. As illustrated in Figure 9, the sensor response to 100 ppm NO_2_ exhibited the highest value compared with other single interfering gas. It was also clearly seen that a slight change in response was observed to the mixture gases of NO_2_, CO and CH_4_, which were mixed in different concentration ratios. However, compared with CO and CH_4_, the sensor response was affected much by the acetone. Actually, as reported in other literature [36,37], LaFeO_3_ was also sensitive to acetone. However, the concentration of acetone in automobile exhaust was lower than NO_2_. The result proved that the presented sensor had good cross-sensitivity to NO_2_.

As the gas sensing mechanism, it could be explained by the mixed potential theory. When the mixed potential sensor Pt/YSZ/LaFeO_3_ exposes to target gases, the electrochemical reactions can occur simultaneously in the TPB. It can be described by the following reactions:

For NO_2_: NO_2_ + e^−^ → NO + O^2−^ (2)

For C_3_H_6_O: C_3_H_6_O + 8O^2−^ → 3CO_2_ + 3H_2_O +16e^−^ (3)

For CO: CO + O^2−^ → CO_2_ + 2e^−^ (4)

For CH_4_: CH_4_ + 4O^2−^ → 2H_2_O + CO_2_ + 8e^−^ (5)

For O_2_: 2O^2−^ ⇋ O_2_ + 4e^−^ (6)

The exchange of ionic (O^2−^) and electronic (e^−^) could form the electrochemical potentials at the two electrodes of the sensor. The dissimilar catalytic activities, disparate gas adsorption and different electrochemical reactions in the YSZ/LaFeO_3_ and the YSZ/Pt interfaces caused different potentials between SE and RE. The electric potential difference between SE and RE was the response V. The potential magnitude of the sensor depended on the catalytic properties between reducing/oxidizing gases and adsorbed gases at the two interfaces. From the equations, it can be easily understood the response to NO_2_ was opposite to that of other interfering gases.

## 4. Conclusions

In this article, the mixed potential NO_2_ sensors based on the porous YSZ layers doped with different concentration graphite were fabricated. The sensor using the porous YSZ layer fabricated by 3 wt% graphite exhibited the highest response (−76.4 mV to 80 ppm NO_2_) at a low operating temperature (250 °C). The sensor also showed good sensitivity to 10–200 ppm NO_2_ and also exhibited superior repeatability and cross-sensitivity. It could be believed this method had a promising prospect because of low-cost, simple process, high sensor response and low power assumption of sensor, which is very attractive for NO_2_ sensing in automobile and industry applications.

## Figures and Tables

**Table 1:** Comparison of the mixed potential NO_2_ type sensors operating temperature.

Sensor Structure	Conc (ppm)	Response (mV)	Temp (°C)	Ref.
NiO/YSZ/Pt	100	106	850	[18]
Nb_2_O_5_/YSZ/Pt	400	35	800	[26]
In_2_O_3_/YSZ/Pt	100	126	700	[16]
CoTaO_6_/YSZ/Pt	100	93	650	[17]
LaFeO_3_/YSZ/Pt	60	45	550	[27]
LaFeO_3_/YSZ/Pt	100	9	550	[28]
Bi_2_W_2_O_9_/YSZ/Pt	100	20	500	[29]
La_0.65_Sr_0.35_MnO_3_/YSZ/Pt	100	48	500	[30]
LaFeO_3_/YSZ/Pt	100	60	450	[31]
SmFeO_3_/YSZ/Pt	90	130	400	[32]
LaFeO_3_/YSZ/Pt	100	164	300	[33]
LaFeO_3_/YSZ/Pt	100	81	250	This work
